# Parasitic plants are one step ahead: *Cuscuta* responds transcriptionally to different hosts

**DOI:** 10.1093/plphys/kiad524

**Published:** 2023-10-04

**Authors:** Manuel González-Fuente

**Affiliations:** Assistant Features Editor, Plant Physiology, American Society of Plant Biologists; Faculty of Biology & Biotechnology, Ruhr-University Bochum, 44801 Bochum, Germany

When we first think about plant pathogens, we usually picture viruses, bacteria, and fungi. What less often comes to mind are other plants. However, parasitic plants are present in all major biomes and can infect many economically important crops, thus posing a threat to food security worldwide. Parasitic plants are greatly diverse, including stem parasites such as mistletoes or dodders, as well as root parasites such as broomrapes, witchweeds, or the famous corpse flower. Despite this large diversity, all parasitic plants share a common specialized organ, the haustorium, that enables them to invade the host and feed from its resources (Yoshida et al. 2016). This parasitic behavior negatively affects the host plant, ultimately leading to severe reductions in crop yields or alterations of forest ecosystems. Therefore, reducing the impact and spread of parasitic plants is crucial. However, as both the parasite and the host are plants, it is quite difficult to develop control strategies that can selectively limit the parasite impact without hindering the host (Fernández-Aparicio et al. 2020). Hence, understanding the molecular mechanisms ruling parasitic-host plant interactions is key to identify and exploit possible specific targets to generate novel strategies to mitigate the effects of parasitic plants. Although some advancements on this front have been made over the years (Jhu and Sinha 2022), we still do not fully understand the complexity of the host defense responses against parasitic plants nor the counteraction mechanisms of the parasite toward the host.

In this issue of *Plant Physiology*, Bawin et al. (2023) conducted dual transcriptomic analyses of both parasite and 2 different host plants with opposing tolerance profiles. This setup allowed them to unveil an intricate transcriptional mechanism of adaption to different defensive strategies of the hosts, particularly at the cell wall composition level. Specifically, the authors worked with the parasitic plant *Cuscuta campestris* (golden dodder) and 2 closely related Solanaceae hosts: the partially resistant *Solanum lycopersicum* (tomato) and its susceptible wild relative *S. pennellii*. Although both hosts allow the successful propagation of *C. campestris*, only *S. lycopersicum* deploys evident defense responses, including necrotic lesions and accumulation of phenolic compounds in the cell wall at the attachment sites.

In a previous work, the authors conducted transcriptomic analyses of *C. campestris* haustoria elegantly induced in the absence of a host by far-red light radiation (Bawin et al. 2022). Comparing these transcriptomes with the current ones extracted from *C. campestris* haustoria at different stages of development in both Solanaceous hosts, the authors observed a major effect of the presence of a host: upon infection of any of the hosts, the number of differentially expressed genes doubled compared with the host-free conditions. Most of these genes were involved in early stages of haustorial development, marked by signatures of cell growth and attachment. Fewer genes were involved in later stages of haustorial development, among which there were genes involved in ethylene biosynthesis, transcription factors, and lytic enzymes, including many cell wall–degrading enzymes. Interestingly, most cell wall–degrading enzyme genes induced during *C. campestris* haustorial development showed increased expression in haustoria infecting the resistant *S. lycopersicum* compared with the susceptible *S. pennellii* and even more elevated compared with the host-free condition. This finding agrees with previous reports pointing to cell wall loosening as a key virulence strategy of dodders (Johnsen et al. 2015).

In light of these results, the authors also examined the host transcriptomes at the site of infection. Among the genes up-regulated exclusively or more strongly in resistant *S. lycopersicum*, there were genes coding for known immune signaling components as well as enzymes involved in synthesis and lignification of the cell wall. This perfectly mirrors the transcriptome of the parasitic plant, as the host seems to reinforce the same structure that the parasite is making an effort to degrade. For instance, if we have a look at mannans, mannose polysaccharides that function as structural and storage components of the cell wall (Voiniciuc 2022), whereas the host (particularly the resistant host) produces mannan synthases upon infection, *C. campestris* produces mannanases (earlier and more strongly when infecting the resistant host). Moreover, the expression of at least one of these mannanases was induced in host-free haustoria treated with certain mannans. This suggests that *C. campestris* might somehow sense the presence of host-derived cell wall compounds and induce the expression of the corresponding degradative enzymes as a response. These observations based on correlative transcriptomic data were elegantly corroborated looking specifically at the presence of mannans in both hosts. In agreement with the different transcriptomic data, resistant *S. lycopersicum* cumulated more mannans at the contact site with *C. campestris* haustoria than in distal non-infected areas. This tendency was not observed in susceptible *S. pennellii*, coinciding with its lack of induction of mannan synthases upon infection.

**Figure 1.**
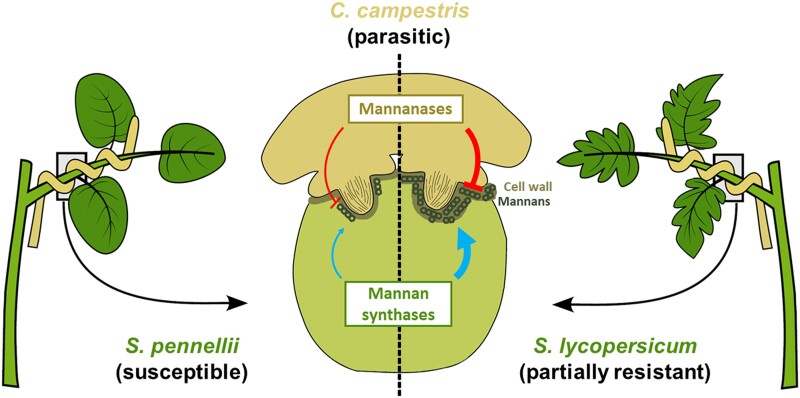
*Cuscuta campestris* adapts to counteract the host reinforcement of the cell wall. Partially resistant host *S. lycopersicum* induces the expression of cell wall reinforcing genes such as mannan synthases upon infection with parasitic plant *C. campestris*; the diagram in the center shows a cross-section of the interaction the parasite and host. The parasite on the other hand, senses host-derived cell wall compounds and counteracts by inducing the expression of cell wall degrading enzymes such as mannanases. These inductions do not occur when the host is susceptible (i.e. *S. pennellii*), revealing the adaptive nature of this mechanism. Figure adapted from Bawin et al. (2023).

In summary, in this study Bawin et al. (2023) dissected the transcriptomic behavior of both parasitic and host plants during different stages of haustorial development and unveiled a mechanism of adaption of *C. campestris* to the defensive cell wall fortification of the host (Fig. 1). Together with previous work (Johnsen et al. 2015; Mitsumasu et al. 2015), this highlights the importance of the cell wall in the establishment of plant parasitism. Therefore, it is not unreasonable to imagine that novel strategies to control the impact of parasitic plants will have their focus on this structure, as it has already been proposed against other types of plant pathogens (Molina et al. 2021).

In addition to cell wall processes, this work also hints at the importance of additional pathways for the host response to the parasite and vice versa. For instance, genes involved in ethylene biosynthesis were induced in both parasite and host. Although the effect of host-derived ethylene in *C. campestris* infection is known (Narukawa et al. 2021), the consequences of the parasite's own production of this phytohormone remain to be studied. Similarly, the induction of genes involved in the catabolism of other hormones such as cytokinins and jasmonic acid in infected hosts was also mentioned by the authors. How or whether these processes are also involved in the response against *C. campestris* and how *C. campestris* potentially deals with it would also require further analyses. Nevertheless, this seminal work lays the groundwork for many future research venues in the field of parasitic-host plant interactions.
